# EGFR–c-Src-Mediated HDAC3 Phosphorylation Exacerbates Invasion of Breast Cancer Cells

**DOI:** 10.3390/cells8080930

**Published:** 2019-08-19

**Authors:** Sung-Min Kwak, Jaesung Seo, Jin-Taek Hwang, Gi-Jun Sung, Ji-Hye Song, Ji-Hoon Jeong, Seung-Hyun Lee, Ho-Geun Yoon, Hyo-Kyoung Choi, Kyung-Chul Choi

**Affiliations:** 1 Department of Biomedical Sciences, Asan Medical Center, AMIST, University of Ulsan College of Medicine, Seoul 05505, Korea; 2 Department of Pharmacology, University of Ulsan College of Medicine, Seoul 05505, Korea; 3 Department of Biochemistry and Molecular Biology, Center for Chronic Metabolic Disease Research, Brain Korea 21 Plus Project for Medical Sciences, Severance Medical Research Institute, Yonsei University College of Medicine, Seoul 03722, Korea; 4 Korea Food Research Institute, Wanju-gun 55365, Korea; 5 Department of Food Biotechnology, Korea University of Science & Technology, Daejeon 34113, Korea

**Keywords:** breast cancer, c-Src, EGFR, HDAC3, pY-HDAC3^Y328/331^ antibody

## Abstract

Breast cancer is one of the leading causes of morbidity and mortality among women. Epidermal growth factor receptor (EGFR) and proto-oncogene tyrosine-protein kinase Src (c-Src) are critical components of the signaling pathways that are associated with breast cancer. However, the regulatory mechanism of histone deacetylase 3 (HDAC3) in these pathways remains unclear. Using the Net Phos 3.1 program for the analysis of kinase consensus motifs, we found two c-Src-mediated putative phosphorylation sites, tyrosine (Tyr, Y)-328 and Y331 on HDAC3, and generated a phospho-specific HDAC3 antibody against these sites. c-Src-mediated phosphorylation was observed in the cells expressing wild-type HDAC3 (HDAC3^WT^), but not in cells overexpressing phosphorylation-defective HDAC3 (HDAC3^Y328/331A^). Phosphorylated HDAC3 showed relatively higher deacetylase activity, and PP2, which is a c-Src inhibitor, blocked HDAC3 phosphorylation and reduced its enzymatic activity. EGF treatment resulted in HDAC3 phosphorylation in both MDA-MB-231 and EGFR-overexpressing MCF7 (MCF7-EGFR) cells, but not in MCF7 cells. Total internal reflection fluorescence analysis showed that HDAC3 was recruited to the plasma membrane following EGF stimulation. HDAC3 inhibition with either c-Src knockdown or PP2 treatment significantly ameliorated the invasiveness of breast cancer cells. Altogether, our findings reveal an EGF signaling cascade involving EGFR, c-Src, and HDAC3 in breast cancer cells.

## 1. Introduction

Breast cancer is the most common malignancy^1^ and the second leading cause of cancer-related deaths among women worldwide [1,2]. In the past, clinical treatments of breast cancer were based on the anatomical extent of the disease, but treatments are now shifting toward addressing the underlying biological mechanisms [2]. Many studies have been performed to evaluate the key molecular signaling pathways involved in breast cancer, but the underlying molecular mechanisms and prospective treatment strategies are incompletely elucidated. A growing body of evidence has implicated the involvement of tyrosine (Tyr, Y) kinases in human breast cancer development [3,4,5,6]. 

The involvement of human epidermal growth factor receptor (EGFR) and c-Src in breast cancer has been examined. The EGF-induced tyrosine phosphorylation of EGFR is caused by the activation of receptor tyrosine kinases or non-receptor tyrosine kinases, including c-Src [7]. EGF-induced EGFR activation results in a physical association between c-Src and EGFR [8,9], the transient activation of c-Src, c-Src-mediated phosphorylation of Y845 on EGFR, and the subsequent stimulation of intracellular pathways that control mitogenic, survival, cell–cell adhesion, migration, and angiogenesis pathways [10,11]. Elevated protein levels and/or the catalytic activity of c-Src have been detected in several human cancers, including lung, skin, colon, breast, ovarian, endometrial, and head and neck cancers [12,13]. In particular, c-Src and EGFR are overexpressed in ~70% of breast cancer cases [5]. In MDA-MB-468 human breast cancer cells, the overexpression of c-Src results in the increase in the phosphorylation of EGFR^Y845^, and cells transiently expressing EGFR with Y845F mutation show EGF-induced inhibition of DNA synthesis [12], indicating that c-Src-mediated EGFR phosphorylation is critical for receptor function and breast cancer cell survival.

HDAC3 belongs to the class I family, wherein HDAC1, HDAC2, and HDAC8 were first identified as the components of the nuclear receptor corepressor (N-CoR) and silencing mediator of retinoic and thyroid receptors (SMRT) corepress complexes [14]. Aberrant expression and/or the localization of HDAC3 was linked with carcinogenesis in various cancers [15]. HDAC3 was recently shown to exhibit high expression in breast cancer, and its expression was significantly correlated with poor overall survival [16]. Although the association with N-CoR/SMRT is a principal regulatory mechanism underlying HDAC3 activity, HDAC3 activity is potentially regulated by reversible phosphorylation/dephosphorylation [17]. However, the regulatory mechanism underlying HDAC3 activity and phosphorylation in the development of breast cancer still remains obscure. In this study, we firstly identified HDAC3 phosphorylation at both Y328 and Y331 residues in response to EGF stimulation in an EGFR-dependent and c-Src-dependent manner, and found that the phosphorylation of HDAC3 significantly enhanced its deacetylase activity, accelerating the invasiveness of breast cancer cells. 

## 2. Materials and Methods

### 2.1. Cell Culture 

Human breast cancer cell lines MCF7 and MDA-MB-231, human embryonic kidney cell line 293T, and the human cervical cancer cell line HeLa were obtained from the American Type Culture Collection (ATCC; Manassas, VA, USA). All the cells were maintained in Dulbecco’s modified Eagle’s medium (DMEM) supplemented with 10% (*v*/*v*) heat-inactivated fetal bovine serum (FBS) and 1% penicillin/streptomycin at 37 °C under 5% CO_2._

### 2.2. Plasmids and Transfections

Wild-type (WT) HDAC3 constructs were generated by standard PCR and cloned in the pSG5-KFM1-Myc, hemagglutinin (HA) (Sigma Aldrich, St. Louis, MO, USA), or pGEX4T-1 (GE healthcare, Piscataway, NJ, USA) vector. 

The full length of c-Src or EGFR was also constructed by PCR and cloned into the pSG5-KFM1-Flag (Sigma Aldrich) vector. To generate HDAC3-C^WT^, the region from 277 aa to 428 aa of HDAC3 was amplified by PCR and subcloned in the pGEX4T-1 (GE healthcare) vector. HA-HDAC3-C^Y328/331A^, shHDAC3-resistant Flag-HDAC3^wt^ (Flag-rsh-HDAC3^WT^), and shHDAC3-resistant Flag-HDAC3^Y328/331A^ (Flag-rshHDAC3^Y328/331^^A^) expression plasmids were derived from HA-HDAC3 or Flag-HDAC using the QuikChange site-directed mutagenesis kit (Stratagene, La Jolla, CA, USA). All of the plasmid constructs were verified by DNA sequencing. Transient transfection was performed using TransIT 2020 (Mirus, Madison, WI, USA) according to the manufacturer’s instruction. To adequate transfection controls, the corresponding empty vectors were used in all of the relevant experiments. 

### 2.3. Prediction of Tyrosine Phosphorylation of HDAC3 and in vitro Kinase Assay 

To predict HDAC3 phosphorylation at tyrosine residues, the Netphos 3.1 server (http://www.cbs.dtu.dk/services/NetPhos/) was used. The protein sequences of HDAC3 (Genebank Accession: AAC52038) were submitted in FAST format, and the results displayed only the best prediction for each residue (Supplementary information Table S1). Glutathione S-transferase (GST)-fusion proteins were expressed in the *Eschericha coli* strain BL21 (DE3) with 0.5 mM of isoprophy-β-d-thiogalactoside for 2 h, and then isolated using glutathione Sepharose 4B beads (GE healthcare) according to the manufacturer’s instruction. GST-HDAC3-C^WT^ and GST-HDAC3-C^Y328/331A^ were incubated with 10 μCi [γ-^32^P] ATP (167 TBq/mmol, ICN Biomedicals, Inc., Irvine, CA, USA), and recombinant c-Src in 30 μL of kinase buffer (25 mM of 4-(2-hydroxyethyl)-1-piperazineethanesulfonic acid (HEPES), 25 mM of β-glycerophosphate, 25 mM of magnesium chloride [MgCl_2_], 2 mM of dithiothreitol [DTT], and 0.1 mM of sodium metavanadate [NaVO_3_]) at 30 °C for 2 h. The reaction was terminated with the addition of Laemmli sodium dodecyl sulfate (SDS) a sample dilution buffer. Proteins were separated by 10% SDS polyacrylamide gel electrophoresis (PAGE), and phosphorylation was visualized by autoradiography. To demonstrate an equal loading of GST-HDAC3 proteins, gels were also stained with Coomassie blue.

### 2.4. Western Blot Analysis and Antibodies

Cells were harvested in phosphate-buffered saline (PBS). Cell extracts were prepared with 150 mM of lysis buffer (50 mM of Tris-Cl [pH 7.4], 150 mM of sodium chloride [NaCl], 1% NP-40, 10 mM of sodium fluoride (NaF), 10 mM of sodium pyrophosphate, and protease inhibitor cocktail [GenDEPOT, Barker, TX, USA]). Lysates were centrifuged at 20,000× *g* for 20 min at 4 °C, and separated by 8–13% SDS-PAGE. The protein bands were transferred onto nitrocellulose membranes (Whatman, Dassel, Germany), and the membranes were blocked in 5% skim milk (BD Biosciences, Sparks, MD, USA) in 1× PBST. The antibody against pHDAC3Y328/331 (αpYHDAC3^Y328/331^) was generated by Abclon (Seoul, South Korea) using the synthetic peptide ^319^ISEELPYSEpYEFpYFAPDFT^337^. Western blots were probed with specific primary antibodies (Table S2), followed by treatment with secondary antibodies. 

### 2.5. Immunoprecipitation (IP) Assay 

Cells were lysed in the lysis buffer as described above. Lysates were clarified by centrifugation at 20,000× *g* for 20 min at 4 °C. The supernatant was collected, and antibody was added. IP was performed with the indicated antibodies (Table S2) and G plus/protein A-agarose bead (Santa Cruz Biotechnology, Dallas, TX, USA). IP assays were incubated overnight with gentle rotation at 4 °C. The beads were washed thrice in a washing buffer for 5 min each and suspended in the electrophoresis sample buffer. Immunoprecipitated proteins were analyzed by Western blotting.

### 2.6. siRNA and Transfection

For siRNA transfection, cells were maintained at 60–70% confluence and transfected using Lipofectamine RNAi MAX (Life Technology, Carlsbad, CA, USA) with 20 pmol following the manufacturer’s protocol. Sequences of small interfering RNAs (siRNAs): sense 5′-CCUCGUGCCGUUCCAUCAGGUAGUU-3′, antisense 5′-CUACCUGAUGGAACGGCACGAGGUU-3′ (Negative control); sense 5′-CAACAAGAUCUGUGAUAUUUU-3′, antisense 5′-AAUAUCACAGAUCUUGUUGUU-3′ (HDAC3); sense 5′-CUGUUCGGAGGCUUCAACU-3′, antisense 5′-AGUUGAAGCCUCCGAACAG-3′ (c-Src); sense 5′-GAUCCACAGGAACUGGAUA-3′, antisense 5′-UAUCCAGUUCCUGUGGAUC-3′ (EGFR #1); sense 5′-GGAUCCACAGGAACUGGAU-3′, antisense 5′-AUCCAGUUCCUGUGGAUCC-3′ (EGFR #2).

### 2.7. Total Internal Reflection Fluorescence Microscopy (TIRF-M) 

Time-lapse imaging was performed by TIRF-M. Cells were cultured on glass coverslips and transfected with enhanced green fluorescent protein (pEGFP) or pEGFP–histone deacetylase 3 (HDAC3). Culture media without phenol red were used during image acquisition, and mineral oil was layered on the top of the media to prevent evaporation. On the day of the experiment, cells transiently expressing control or GFP-HDAC3 were serum-starved for 1 h and then treated with 10 ng/mL of EGF during imaging. After 10 s of ligand addition, time-lapse images were acquired at a frame rate of 1/s and an exposure time of 80–150 ms using an Eclipse TE2000 (Nikon, Kanagawa, Japan)

### 2.8. Cell Fractionation

Cells were washed twice with PBS, trypsinized, and centrifuged at 1000 *g* for 3 min at 4 °C. The harvested cells were resuspended in 1 mL of solution I buffer (10 mmol/L Tris (pH 7.4), 10 mmol/L of KCl, and 3 mmol/L of MgCl_2_, 0.5% NP-40) containing protease inhibitor cocktail; then, cells were incubated for 20 min on ice and centrifuged at 1500× *g* for 5 min at 4 °C. The supernatants were transferred into another tube (cytosol fraction), and the pellet was resuspended in 200 μL of solution II buffer (20 mmol/L of Tris (pH 7.9), 0.42 mol/L of NaCl, 0.2mmol/L of EDTA, 10% glycerol, 2 mmol/L of Dithiothreitol (DTT)) containing protease inhibitor cocktail using five strokes of a syringe. The suspension was incubated for 20 min on ice and centrifuged at 17,000 *g* for 20 min for nuclear faction. To take the membrane fraction into account, a subcellular protein fraction kit (Thermo Fisher Scientific, Rockford, IL, USA) was used according to the manufacture’s protocol. Briefly, the cell pellet was incubated with cytosol extraction buffer at 4 °C for 10 min, after which the lysates were centrifuged at 500× *g* for 5 min, and then immediately transferred to the supernatant (cytoplasmic extract). Next, ice-cold membrane extraction buffer containing protease inhibitor cocktail was added into the left pellet and vortexed thoroughly; then, the pellet was incubated at 4 °C for 15 min. Finally, the lysates were centrifuged at 3000× *g* for 5 min, and the supernatant was used for membrane protein to carry out IP.

### 2.9. Immunofluorescence Analysis

MDA-MB-231 cells were plated at a density of 1 × 10^5^ cells per 22-mm glass coverslip for 24 h before EGF treatment. After incubation with EGF for 24 h, cells were fixed for 30 min in cold 4% paraformaldehyde and permeabilized for 2 min at 25 °C in 0.25% Triton X-100. Expressed HDAC3 was detected by incubation at 4 °C with an anti-HDAC3 antibody. After three washes with PBS, coverslips were incubated for 1 h at 37 °C with goat anti-rabbit IgG (H+L) secondary antibody conjugated rhodamine (ThermoFicher Scientific, Waltham, MA, USA). Coverslips were mounted in a medium containing 4′,6-diamino-2-phenylindole (DAPI). Cells were examined with a ZOE Fluorescent Cell imager (BIO-RAD, Hercules, CA, USA).

### 2.10. Assay for HDAC3 Activity

The activity of HDAC3 was measured using an HDAC3 assay kit (Biovision, Milpitas, CA, USA) according to the manufacturer’s protocol. Cell lysates were immunoprecipitated with anti-HA or anti-HDAC3 antibody and prepared for HDAC3 assay. A relative fluorescence unit was measured using a microplate reader (Molecular Devices, San Jose, CA, USA).

### 2.11. Matrigel Invasion Assays 

The in vitro invasiveness of cells was evaluated by determining the ability of cells to transmigrate through a layer of extracellular matrix in Biocoat Matrigel invasion chambers (SPL Life Science, Pocheon, Korea). HDAC3 or c-Src siRNA was transiently transfected in MDA-MB-231 cells, and cells were seeded at a density of 2.0 × 10^4^ cells per insert and cultured for 24 h. The cells were placed in wells containing the same medium supplemented with 10 ng/mL of EGF with or without PP2 (10 μM). After 2 h, non-invading cells were removed with cotton swabs. Invading cells were fixed with 100% methanol and stained with 1% crystal violet (Sigma-Aldrich) before enumeration under an inverted microscope (40×, three random fields per well). Data are expressed as the mean ± standard deviation (SD) of at least three independent experiments. 

### 2.12. Lentiviral shRNAs

For the stable knockdown of HDAC3 gene expression, two pairs of oligonucleotides (5′–3′) that encoded short hairpin RNA (shRNA) against target MISSION shRNA were purchased (Sigma-Aldrich). To generate lentiviral particles, pLKO.1-PURO HDAC3 plasmid with three plasmids (pMDLg/pRRE, envelope RSV-REV and pMD2.G) were co-transfected using Lipofectamine 3000 (Invitrogen, Grand Island, NY, USA) in a 293FT cell line. After 72 h of incubation, supernatants were collected and filtered using a 0.45-µm pore. Then, MDA-MB-231 cells were infected with lentivirus particles. After incubation with virus supernatant for 2 days, cells were selected with 1 µg·mL^−1^ of puromycin (Sigma-Aldrich). Lentivirus PURO shRNA was generated as a control. 

### 2.13. Statistical Analysis

Statistical significance was examined using Student’s *t*-test. Two-sample *t*-test was used for two-group comparisons. Values are reported as mean ± SD. A value of *P* < 0.05 was considered significant.

## 3. Results

### 3.1. Phosphorylation of HDAC3 at Tyrosine

**Figure 1:**
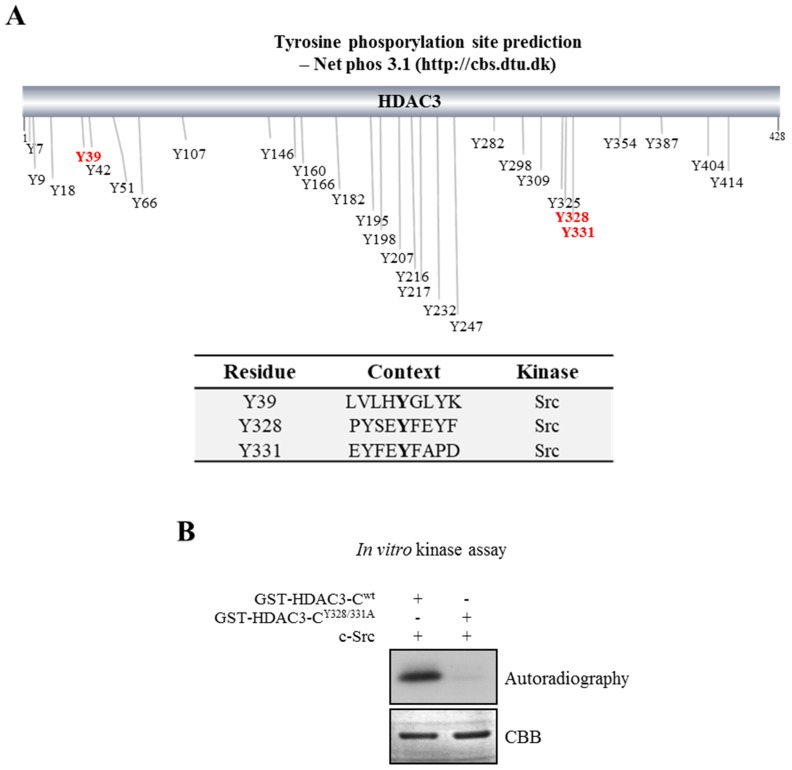
In vitro phosphorylation of Y328 and Y331 on histone deacetylase 3 (HDAC3). (**A**) Prediction of putative phosphorylation sites at tyrosine residues on HDAC3. Putative tyrosine phosphorylation residues on HDAC3 were predicted using an artificial neural network predictor of phosphorylation sites, the Net Phos3.1 (http://cbs.dtu.dk) program. The proto-oncogene tyrosine-protein kinase (c-Src)-mediated putative phosphorylation residues are highlighted in red. (**B**) HDAC3 is phosphorylated by c-Src. In vitro kinase assays were performed with recombinant c-Src and the indicated glutathione (GST)-fused HDAC3 proteins. The samples were processed for SDS-PAGE and subsequently visualized by autoradiography. CBB, Coomassie blue staining.

To determine the amino acid residues that are essential for the c-Src-mediated phosphorylation of HDAC3, we used an artificial neural network predictor of phosphorylation sites, the Net Phos3.1 (http://cbs.dtu.dk) program [18], for sequence analyses to predict putative phosphorylation sites on HDAC. As shown in Figure 1A and Table S1 (each residue displays only the best prediction), three putative phosphorylation sites, Y39, Y328, and Y331, were screened as the target residues on HDAC3 for c-Src kinase. The post-translational modifications on HDAC3 that affected its activity, including phosphorylation at S424 and cleavage at D391, were reported to occur around its C-terminal region (HDAC3-C) [19,20,21]. Moreover, the Y298 residue on HDAC3-C is known to play a key role in HDAC3 activity [22]. Based on these previous reports, we thought that Y328 and Y331 on HDAC3-C would be more important than Y39 for its activity regulation. To determine whether HDAC3 was phosphorylated at Y328 and Y331 by c-Src, an in vitro kinase assay was performed using GST-tagged wild-type HDAC3-C (HDAC3-C^WT^) and mutant HDAC3-C^Y328/331A^ with tyrosine substituted for alanine (Ala, A). As shown in Figure 1B, c-Src efficiently phosphorylated GST-HDAC3-C^WT^ but not the mutant protein, GST-HDAC3-C^Y328/331A^, resulting in the complete loss of phosphorylation. This observation suggests that Y328 and Y331 residues of HDAC3 are critical for c-Src-dependent phosphorylation.

### 3.2. c-Src Phosphorylates Y328 and Y331 on HDAC3

**Figure 2:**
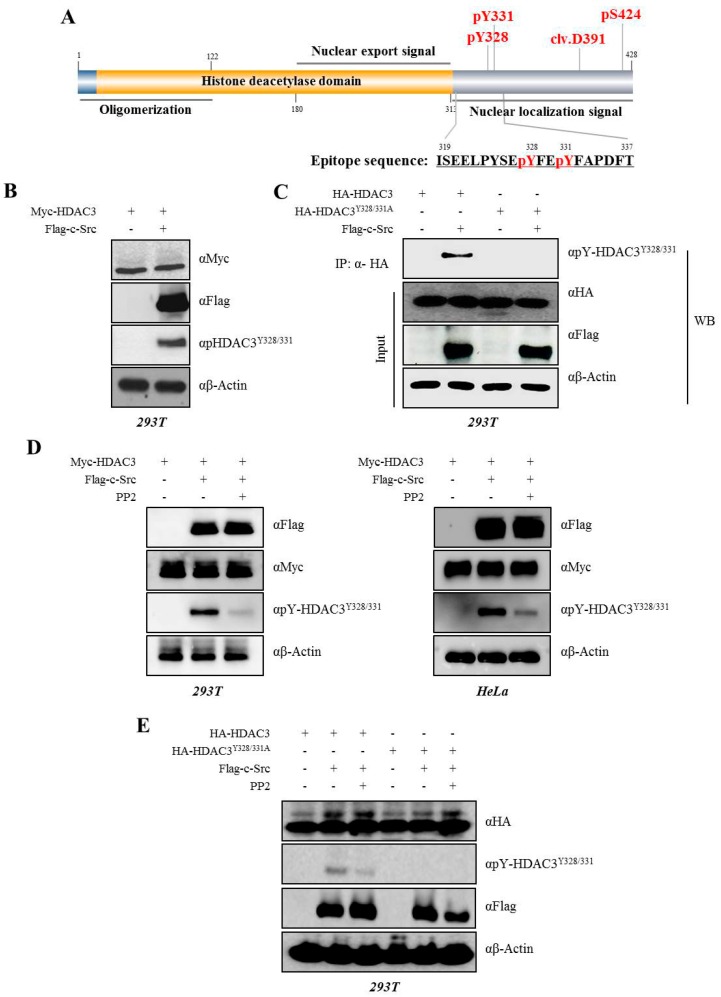
c-Src phosphorylates Y328 and Y331 on HDAC3 in 293T cells. (**A**) Modified peptide sequence for the generation of phospho-HDAC3^Y328/331^ antibody. Schematic diagram of human HDAC3 protein with multiple functional domains. Residues 1 to 122 are necessary for HDAC3 oligomerization. The actual functional nuclear export signal and nuclear localization signal reside between residues 180–313 and 313–428, respectively. pS424, the phosphorylation site of serine 424 on HDAC3; clv.D391, cleavage site of aspartate 391 on HDAC3. (**B**) c-Src phosphorylates HDAC3 in 293T cells. Myc-tagged HDAC3 was transiently transfected with/without Flag-tagged c-Src in 293T cells. After 48 h, cells were harvested and cell lysates were analyzed by Western blotting with indicated antibodies. (**C**) Y328 and Y331 are important for the c-Src-mediated phosphorylation of HDAC3. Either HA-HDAC3^WT^ or HA-HDAC3^Y328/331A^ plasmid was transfected with/without Flag-c-Src into 293T cells. After 48 h, various HDAC3 plasmid samples were immunoprecipitated with HA antibody and analyzed by Western blotting with indicated antibodies. (**D**) The c-Src-mediated phosphorylation of Y328 and Y331 on HDAC3 was interfered by PP2, which is a c-Src inhibitor. Myc-HDAC3 was transfected with/without Flag-c-Src into 293T (left panels) and HeLa (right panels) cells, followed by the treatment of cells with 10 µM of PP2 for 30 min before harvest. Cell lysates were analyzed by Western blotting with indicated antibodies. (**E**) Either HA-HDAC3^WT^ or HA-HDAC3^Y328/331A^ plasmid was transfected with/without Flag-c-Src into 293T cells, followed by the treatment of cells with PP2, a c-Src inhibitor, at 10-µM concentration for 30 min before harvest. Cell lysates were analyzed by Western blotting with indicated antibodies.

We confirmed the c-Src-mediated phosphorylation of Y328 and Y331 on HDAC3 with the generated phosphor HDAC3^Y328/331^ antibody (αpHDAC3Y^328/331^) with a modified peptide from amino acids 319 to 337 serving as an epitope (Figure 2A) using an IP assay. As shown in Figure 2, the phosphorylation of Myc-HDAC3^WT^ was observed after the co-transfection with Flag-c-Src in 293T cells (Figure 2B). On the other hand, we did not report phosphorylation at Y328 and Y331 in cells overexpressing the mutant HA-HDAC3^Y328/331A^ (Figure 2C), indicating that these are c-Src-mediated phosphorylation sites. To confirm that HDAC3 phosphorylation was strongly dependent on c-Src, we treated cells with PP2, which is a selective inhibitor of c-Src. 293T and HeLa cells were co-transfected with Myc-HDAC3 alone or in combination with Flag-c-Src with/without PP2 (Figure 2D, left or right panel, respectively). The phosphorylation of HDAC3 was detected in cells overexpressing Flag-c-Src, but was inhibited in cells treated with PP2. We also expressed HDAC3^Y328/331^ in a similar manner (Figure 2E).

### 3.3. c-Src-mediated Phosphorylation of HDAC3 at Y328 and Y331 Alters HDAC3 Enzymatic Activity

**Figure 3:**
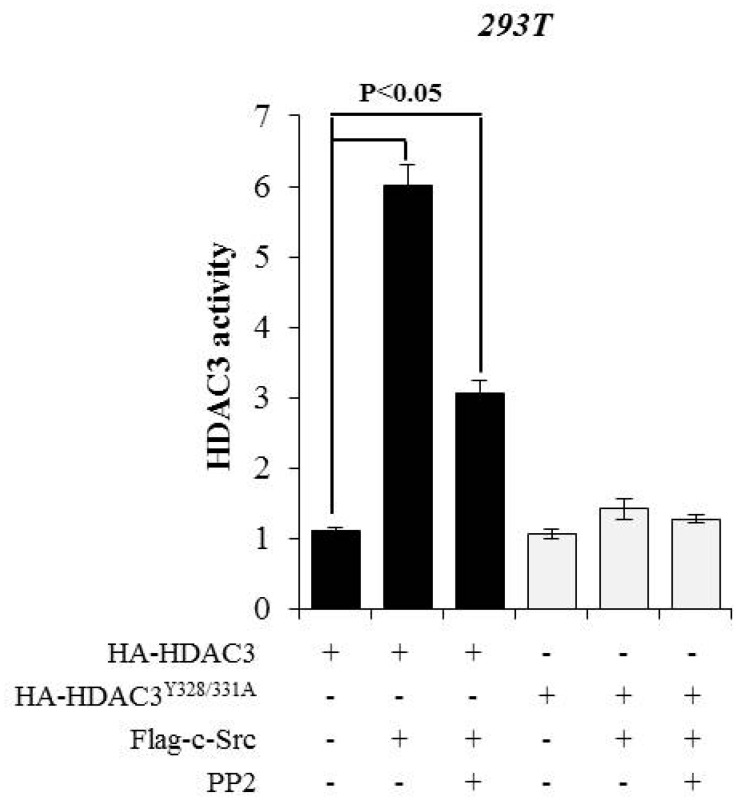
c-Src-mediated phosphorylation of HDAC3 affects its enzymatic activity. Mutations of Y328 and Y331 on HDAC3 inhibited its enzymatic activity. 293T cells were transfected with plasmids encoding either HA-tagged HDAC3^WT^ or HA-tagged HDAC3^Y328/331A^. Cell extracts were immunoprecipitated with anti-HA antibody and assayed for histone deacetylase (HDAC) activity. The results were expressed as fold changes in optical density (OD) values relative to OD values of control (HA-HDAC3-overexpressing cells). The values presented are the means ± SD of three independent experiments.

Previous studies have suggested that the phosphorylation status of class I HDACs, including HDAC1, HDAC2, or HDAC8, affects their enzymatic activities [17,23,24]. Furthermore, the phosphorylation of S424 on HDAC3 was shown to influence its enzymatic activity [21]. To determine whether the phosphorylation of Y328 and Y331 on HDAC3 is associated with its enzymatic activity, we expressed HA-tagged HDAC3^WT^ or HDAC3^Y328/331A^ in combination with c-Src with/without PP2 in 293T cells and immunoprecipitated the proteins with HA antibody to measure the deacetylase activity. As shown in Figure 3, the histone deacetylase activity of HDAC3 was dependent on its phosphorylation status. HDAC3^WT^ in combination with c-Src showed a dramatic increase in the enzymatic activity (*P* < 0.05), which was reduced following PP2 treatment. On the other hand, HDAC3^Y328/331^ mutant was not phosphorylated by c-Src, and hence failed to show any increase in the enzymatic activity, even in the presence of c-Src overexpression. Altogether, these data suggest that the c-Src-mediated phosphorylation of HDAC3 at Y328 and Y331 is critical for its enzymatic activity.

### 3.4. EGFR is Essential for c-Src-mediated HDAC3 Phosphorylation

**Figure 4:**
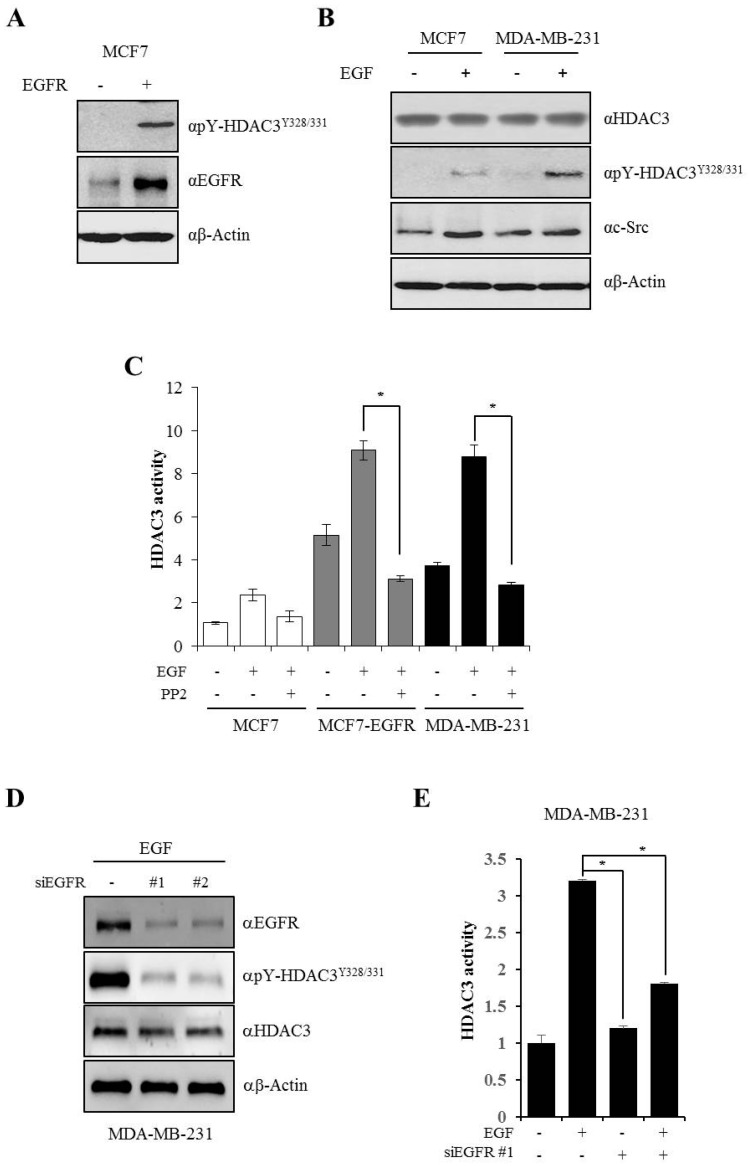
Epidermal growth factor (EGF)-induced c-Src-mediated phosphorylation of HDAC3 is dependent on epidermal growth factor receptor (EGFR). (**A**) EGFR is essential for the phosphorylation of HDAC3. EGFR was transfected into MCF7 cells, and cell lysates were analyzed by Western blotting with indicated antibodies. (**B**) EGF-induced HDAC3 phosphorylation is dependent on EGFR. MCF7 and MDA-MB-231 cells were treated with EGF, and cell lysates were analyzed with indicated antibodies. (**C**) The activation of EGFR signaling is critical for HDAC3 enzymatic activity. Various breast cancer cells were treated with EGF with/without PP2. Cell extracts were immunoprecipitated with anti-HDAC3 antibody and assayed for histone deacetylase (HDAC) activity. (**D**) The phosphorylation of HDAC3^Y328/331^ is dependent on the EGFR signaling pathway. Phosphor-HDAC3^Y328/331^ was observed by Western blotting after EGFR knockdown under the indicated condition in MDA-MB-231 cells. (**E**) HDAC3 activity is dependent on EGFR. Small interfering RNA against EGFR (siEGFR) was transfected, and EGF was treated for 20 min before harvest. Cell extracts were immunoprecipitated with anti-HDAC3 antibody and assayed for HDAC activity. Results are expressed as fold changes in OD values relative to OD values of control. The values presented are the means ± SD of three independent experiments. *P ≤ 0.05.

Studies have indicated that EGFR and c-Src contribute to the aggressive phenotype in various human cancers, especially breast cancer. Cells expressing high levels of EGFR and c-Src show dramatic increase in their growth and malignant properties upon continuous exposure to EGF [9,25]. To evaluate whether EGF-induced EGFR-c-Src activation is critical for the phosphorylation of Y328 and Y331 on HDAC3 and determine its association with the malignant behavior of cancer cells, we transiently transfected EGFR in MCF7 cells, which express low levels of EGFR. Y328 and Y331 phosphorylation on HDAC3 was observed in EGFR-transfected cells even without EGF stimulation (Figure 4A). Next, we compared EGF-induced HDAC3 phosphorylation in both MCF7 and MDA-MB-231 cells. Although c-Src expression was more stabilized by EGF in MCF7 cells than in MDA-MD-231 cells, HDAC3 phosphorylation was higher in MDA-MB-231 cells than in MCF7 cells in response to EGF stimulation; no changes were observed in the total expression levels of HDAC3 in both cells (Figure 4B). Next, to explore whether EGFR-c-Src-mediated HDAC3 phosphorylation affects the enzymatic activity of HDAC3, we measured HDAC3 activity with IP assay using endogenous HDAC3 antibody in MCF7, EGFR-overexpressing MCF7 (MCF7–EGFR), and MDA-MB-231 cells. As shown in Figure 4C, HDAC3 activity was higher in both MCF7–EGFR and MDA-MB-231 cells than in MCF7 cells in the absence of EGF stimulation, and the enzymatic activity was dramatically increased following EGF treatment in MCF7–EGFR and MDA-MB-231 cells. However, in both cell lines, an EGF-induced increase in HDAC3 enzymatic activity was completely blocked following the PP2-mediated inhibition of c-Src expression. To confirm that the phosphorylation and activity of HDAC3 is dependent on the EGFR signaling pathway, we observed the phosphorylation of HDAC3^Y328/331^ and HDAC3 activity after the knockdown of EGFR. As expected, the phosphorylation of HDAC3^Y328/331^ was dramatically decreased by siEGFR in the presence of EGF (Figure 4D). Also, HDAC3 activity was weakened following siEGFR treatment (Figure 4E). Taken together, EGF-induced c-Src-mediated Y328 and Y331 phosphorylation on HDAC3 is dependent on EGFR and is strongly associated HDAC3 enzymatic activity.

### 3.5. EGF-Induced Phosphorylation of HDAC3 Affects Its Localization

**Figure 5:**
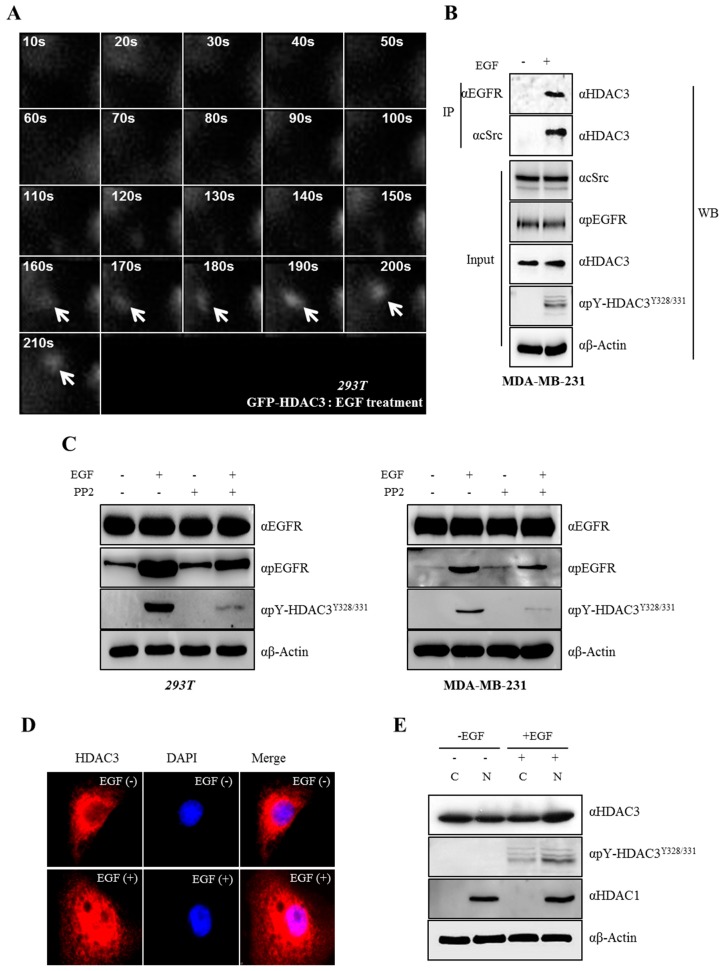
Epidermal growth factor (EGF) triggers phosphorylation and induces the relocalization of HDAC3. (**A**) HDAC3 is recruited to the c-Src–EGFR complex for c-Src-mediated phosphorylation upon EGF treatment. The enhanced green fluorescence protein (EGFP)–HDAC3 plasmid was transfected into 293T cells, followed by the treatment of cells with EGF. The GFP–HDAC3 single molecule was visualized using total internal reflection fluorescence (TIRF) microscopy. Images were captured at every minute for 210 s. Arrows indicate the HDAC3 molecule. (**B**) EGFR–c-Src–HDAC3 forms complexes in the presence of EGF. EGF was treated for 20 min in MDA-MB-231 cells, membrane protein was extracted following the manufacturer’s instruction (see materials and methods), and then immunoprecipitation was carried out with the indicated antibodies. (**C**) EGF induces the phosphorylation of Y328 and Y331 on HDAC3. 293T (left panel) or MDA-MB-231 cells (right panel) were treated with EGF in the presence or absence of PP2 for 20 min. Cell lysates were analyzed by Western blotting with indicated antibodies. (**D**) EGF-induced phosphorylation of HDAC3 is required for its nuclear translocation. Cells were treated with EGF for 20 min. Immunofluorescence analysis was performed. (**E**) Phosphorylation of HDAC3^Y328/331^ affects the translocation of HDAC3. Cells were treated with EGF for 20 min, and then fractionized into the cytosol and nucleus; then, Western blot assays were performed with the indicated antibodies. HDAC1 was used as a positive control for the nuclear fraction.

Above all, we tested whether HDAC3 is recruited to the c-Src–EGFR complex for c-Src-mediated phosphorylation following EGF treatment. The localization of HDAC3 was tracked through TIRF-M. The signal of HDAC3 was detected near the plasma membrane after 160 s, and was found closest to the membrane after 190 s of EGF treatment, suggesting that HDAC3 rapidly moved to the plasma membrane upon EGF treatment (Figure 5A). Indeed, to explore whether EGFR–c-Src-HDAC3 forms complexes in the presence of EGF, we carried out IP assay with a membrane fraction. As shown in Figure 5B, EGFR–c-Scr-HDAC3 was successfully associated following EGF treatment. To investigate the functional consequence of c-Src-mediated HDAC3 phosphorylation, we observed whether the phosphorylation of Y328 and Y331 on HDAC3 was endogenously triggered by EGF-induced c-Src activation. As expected, HDAC3 phosphorylation at two sites was observed in EGF-treated cells, but was completely blocked following the PP2-mediated inhibition of c-Src (Figure 5C). As the two phosphorylation sites of HDAC3 were localized in the nuclear localization signal (NLS) (Figure 2A), we evaluated whether c-Src-mediated HDAC3 phosphorylation affects its cellular localization. c-Src and EGFR have been shown to form an EGF-dependent heterocomplex [26]. Following EGF stimulation, cells were immunostained with HDAC3 antibody and rhodamine and examined by confocal microscopy. As shown in Figure 5D, HDAC3 was localized in the cytoplasm in the absence of EGF, but mainly observed in the nucleus in the presence of EGF. Finally, to prove that HDAC3 phosphorylation at Y328/331 affects its translocation after EGF stimulation, we carried out Western blot analysis after cellular fractionation. The phosphorylation of HDAC3Y328/331 was detected in both cytosol and nuclear following EGF treatment, and the expression of HDAC3 was shown to be the same with the result of immuostaining (Figure 5E), suggesting that the EGFR–c-Src-mediated phosphorylation of Y328 and Y331 is required for the nuclear translocation of HDAC3 in response to EGF stimulation.

### 3.6. EGFR–c-Scr-mediated HDAC3 Phosphorylation Is Crucial for the Invasion of Breast Cancer Cells

**Figure 6:**
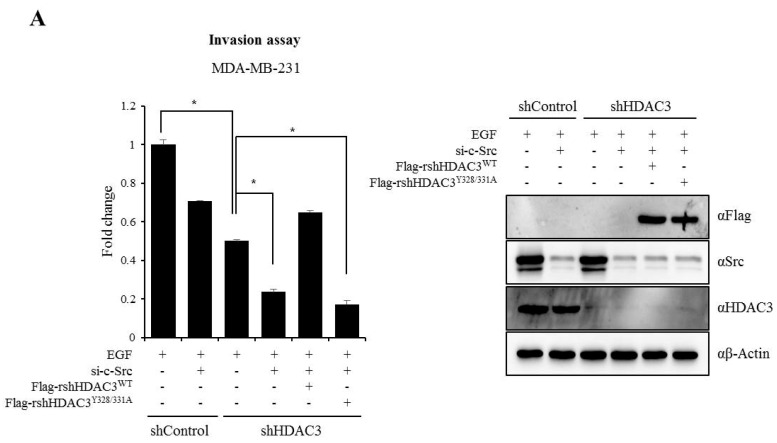

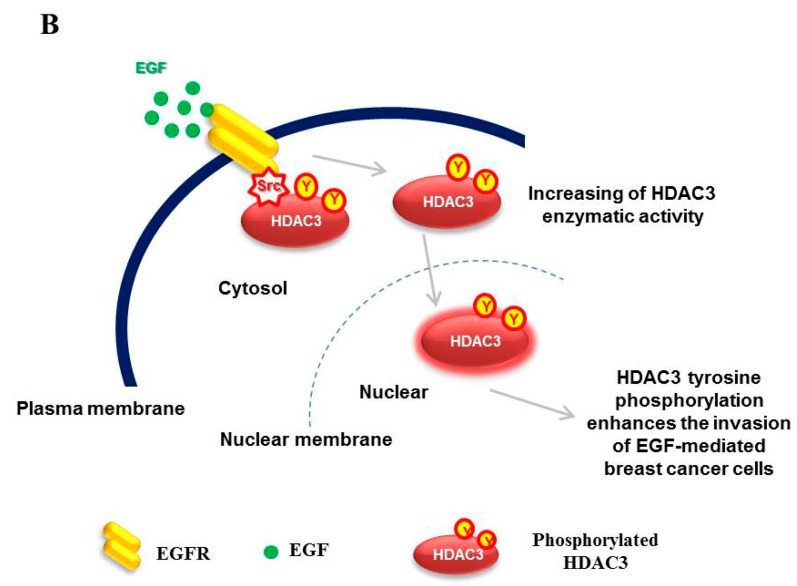
c-Src-mediated HDAC3 phosphorylation enhances the invasion of breast cancer cells. (**A**) siRNA targeting c-Src was transiently transfected into either the sh-Control or sh-HDAC3-expressed stable MDA-MB-231 cells, and after 24 h, rsh-HDAC3^WT^ or rsh-HDAC3^Y328/331^ was overexpressed for another 36 h. Cell invasion was measured using the Matrigel system, and results are presented as the fold of the control (left panel). Results are expressed as means ± SD calculated from three independent experiments. *P ≤ 0.05. Small interfering RAN against c-Src (si-c-Src), Flag-rsh-HDAC3^wt^, and Flag-rsh-HDAC3^Y328/331^ were validated with indicated antibodies using Western blot analysis (right panel). (**B**) Model of our findings. EGF stimulation triggers the formation of the EGFR–c-Src–HDAC3 heterocomplexes near the plasma membrane, induces the EGFR–c-Src-mediated phosphorylation of Y328 and Y331 on HDAC3, resulting in the induction of HDAC3 enzymatic activation. Phosphor-HDAC3Y328/331 was translocated into the nucleus. The association of EGFR–c-Src-HDAC3, consequentially, enhanced the invasiveness of breast cancer cells.

Finally, to confirm whether EGFR–c-Src-mediated HDAC3 phosphorylation was critical for the invasion of breast cancer cells, we performed rescue experiment using shRNA-resistant HDAC3 plasmids (rsh-HDAC3). rsh-HDAC3^WT^ or rsh-HDAC3^Y328/331A^ was transiently transfected into the sh-HDAC3 infected stable MDA-MB-231 cells after the knockdown of c-Src, and cell invasion ability was measured using the Matrigel system following EGFR stimulation. As shown in Figure 6A, the invasiveness of breast cancer cells decreased in the c-Src knocked down sh-Control and sh-HDAC3 infected cells. Also, it was shown to have relatively strong inhibitory effect in c-Src knocked downed sh-HDAC3-infected stable cells. Interestingly, decreased invasiveness following the knockdown of c-Src and HDAC3 was re-increased to the level of that of the sh-Control cells transiently transfected si-c-Src. However, rshHDAC3^Y328/331^ did not rescue the invasion ability of breast cancer cells, indicating that EGFR–c-Src-mediated HDAC3 phosphorylation is involved in the invasion of breast cancer cells. Taken along with our previous results, HDAC3 phosphorylation by EGFR–c-Src may be involved in regulating the invasiveness of breast cancer cells through the modulation of its enzymatic activity.

## 4. Discussion

Histone deacetylase 3 belongs to the class I HDAC family, which includes HDAC1, HDAC2, HDAC3, and HDAC8 [27]. Class I HDACs commonly have a huge impact on the development and progression of various cancers. Several studies have highlighted HDAC overexpression in certain cancer types [28,29,30]. Thus, HDAC inhibition may lead to the attenuation of malignant factors such as growth, migration, invasion, and apoptosis [31]. Although the association with the expression of class I HDACs is principally involved in the regulation of HDAC enzymatic activity, HDAC3 phosphorylation is also thought to play an important role in the regulation HDAC3 enzyme activity [17,21].

In the present study, we observed the c-Src-mediated phosphorylation of Y328 and Y331 on HDAC3 (Figure 1 and Figure 2) and demonstrated that the phosphorylation status of HDAC3 correlated with its deacetylase activity (Figure 3 and Figure 4). HDAC3 is primarily phosphorylated at its unique S424 residue [19,21], unlike HDAC1 and HDAC2, which are phosphorylated at multiple residues [24,32]. We first identified the phosphorylation of HDAC3 on residues other than S424. The proto-oncogene c-Src, which is the most widely studied member of the largest family of the non-receptor protein kinases, has been implicated in the tumorigenesis of breast cancer [33]. The relationship between HDAC3 and breast cancer has been recently reported. A previous study examined the expression of HDAC3 in 145 patients with ductal breast cancer by tissue microarray and showed that HDAC3 expression was associated with clinicopathological factors and the prognostic significance of breast cancer in these patients [16]. Furthermore, an HDAC3-selective inhibitor was shown to suppress the growth of triple-negative breast cancer stem cells in vitro and in vivo [34]. These reports provided the firm evidence of the correlation of c-Src and HDAC3 with the signaling pathway involved in the development and progression of breast cancer.

Class I HDAC phosphorylation and enzymatic activity are strongly correlated. The phosphorylation status of HDAC3 is critical for the regulation of its deacetylase activity [17,20,21]. The phosphorylation of HDAC3 was shown to increase its enzyme activity. Consistent with this result, the c-Src-mediated phosphorylation of Y328 and Y331 on HDAC3 positively increased its enzymatic activity, which was inhibited following c-Src inhibition by PP2 (Figure 3 and Figure 4C). It is suggested that the phosphorylation of HDAC3 may cause a conformational change, leading to a more active conformation. Zhang et al. showed that P-element induced wimpy testis-like (PIWIL)-2 stabilizes HDAC3 by inhibiting E3 ubiquitin-ligase seven in absentia homolog 2 -mediated degradation through casein kinase 2-mediated HDAC3 phosphorylation, thereby accelerating the proliferation of p53-positive cancer cells, followed by a decrease in p21 expression [35]. Another study demonstrated that the phosphatase and tensin homolog-induced putative kinase 1-mediated phosphorylation of HDAC3 results in the suppression of dopaminergic neuronal cell death through an increase in the deacetylase activity via blockage of the HDAC3 cleavage in response to oxidative stress [19]. The cleavage of HDAC3 was closely related to its stability [20]. The difference in the ability of the phosphorylated versus non-phosphorylated HDAC3 to associate with the N-CoR/SMRT corepressor complex is not investigated; thus, it is possible that the phosphorylated HDAC3 may stabilize the quaternary structure of the N-CoR/SMRT corepressor complex as phosphorylated HDAC3 is imported to the nucleus (Figure 5B).

In 2006, researchers demonstrated the localization of HDAC3 to the plasma membrane and its function as a substrate of c-Src [36]. c-Src phosphorylated the nuclear/cytoplasmic HDAC3, and Tyr-phosphorylated HDAC3 was located at the plasma membrane. However, the authors did not identify the tyrosine residue of HDAC3 that was targeted by c-Src. In line with these results, HDAC3 was shown to rapidly move to the plasma membrane following EGF-induced c-Src activation in TIRF images (Figure 5A) and formed complexes with EGFR–c-Src (Figure 5B); however, whether c-Src-mediated HDAC3 phosphorylation occurred in the nucleus/cytoplasm or in the membrane after its movement was not verified in this study. How the phosphorylation of Y328 and Y331 on HDAC3 changes the subcellular localization of HDAC3 is questionable. As shown in Figure 5D,E, HDAC3 was primarily detected in the nucleus following EGF stimulation. Several studies have shown that phosphorylation within an NLS results in the upregulation of the nuclear import in several different ways such as by [37] (i) enhancing the binding affinity for the isoform importin proteins [38]; (ii) enhancing docking cargos to the nuclear pore complex [39,40]; (iii) inducing conformational changes [41,42]; (iv) unmasking the NLS (nucleus localization sequence) through the disruption of the NES (nucleus export sequence) [43]; and (v) activating non-canonical transport signals that mediated nuclear import [44,45]. It was shown that the mutation of specific phosphorylation sites of HDACs decreased the binding affinity with importin-α, resulting in the poor efficiency of nuclear imports [46,47]. In addition, it was found that leucine-rich repeat kinase 2 directly phosphorylates HDAC3 at S424 and stimulates the nuclear translocation of HDAC3 through the phosphorylation of the karyopherin subunit α2 and α6, which are involved in the transport of the molecules between the cytoplasm and nucleus [48]. Although we did not elucidate the exact mechanism involved in the import of HDAC3 in the nucleus following EGF treatment, previous findings strongly support the possibility that the molecules involved in protein import, such as importins or karyopherins, may be engaged in the translocation of EGF-induced tyrosine phosphorylation of HDAC3.

The protein EGFR is a representative cooperator of c-Src in a regulatory mechanism of breast cancer. In the present study, c-Src-mediated tyrosine phosphorylation of HDAC3 was dependent on EGFR expression. Phospho-HDAC3 was undetected in MCF7 cells, which show low EGFR expression, and was rescued by the exogenous overexpression of EGFR (Figure 4A). EGF-induced tyrosine phosphorylation was also observed in MDA-MB-231 cells expressing EGFR (Figure 4B). EGF stimulation was shown to strongly activate EGFR with the phosphorylation of Y992, Y1045, Y1068, Y1086, and Y1173 [49]. In particular, Y1068 and Y1173 residues are thought to be involved in autophosphorylation [50]. Activated EGFR forms a heterocomplex with c-Src [9] and increases c-Src activity, while the activated c-Src [51] sequentially phosphorylates Y845 of EGFR [26]. Hence, both EGFR and c-Src enhance the effects of one another. Our data demonstrated that HDAC3 was rapidly recruited to the plasma membrane following EGFR stimulation. These facts suggest that EGFR is responsible for HDAC3 phosphorylation and activation (Figure 4D,E). Aside from EGFR-c-Src, HDAC3 may be critically involved in the malignant behavior of breast cancer. As shown in Figure 6A, the invasiveness of MDA-MB-231 cells was strikingly decreased (almost 50%) in response to HDAC3 knockdown by shHDAC3 lentiviral infection. Furthermore, the knockdown of both c-Src and HDAC3 was shown to synergistically inhibit the invasion of MDA-MB-231 cells. Strikingly, the decreased invasiveness was re-increased by the transient transfection of the shRNA-resistant HDAC3^wt^ plasmid, but not by rshHDAC3^Y321/331^, strongly confirming that EGFR–c-Src mediated HDAC3 phosphorylation at Y321 and 331 was crucial for the invasion of breast cancer cells. At the same time, our results support the previous reports, highlighting the importance of HDAC3 selective inhibition in the regulation of breast cancer [16,34,52].

Taken together, the present study shows that EGF-induced EGFR activation triggers the c-Src-mediated phosphorylation of Y328 and Y331 on HDAC3. The activated (phosphorylated) HDAC3 promotes the invasion of MDA-MB-231 cells (Figure 6B). Our findings identify the activation mechanism of HDAC3 and propose a new therapeutic target for the treatment of breast cancer. However, the present study has a few limitations. The present study has not indicated the exact molecular mechanism involved in either the changes in the subcellular localization of HDAC3 or the regulation of invasion of breast cancer cells. Given the importance of the aforementioned limitations, further relevant in-depth studies are desirable.

## Supplementary Materials

The following are available online at https://www.mdpi.com/2073-4409/8/8/930/s1. Table S1. Results of computational prediction for HDAC3 tyrosine phosphorylation. Table S2. Antibodies used for Western blotting and Immunoprecipitation assays.

## Abbreviations

HDAC3, histone deacetylase 3; EGFR, epidermal growth factor receptor; N-CoR, nuclear receptor corepressor; SMRT, silencing mediator of retinoic and thyroid receptors; HA, hemagglutinin; TIRF-M, total internal reflection fluorescence microscopy; NLS, nuclear localization signal.
