# Supplementary material for: EGFR–c-Src-Mediated HDAC3 Phosphorylation Exacerbates Invasion of Breast Cancer Cells
Source: Cells. 2019 Aug 19;8(8):930. doi: 10.3390/cells8080930 (PMC6721651; doi:10.3390/cells8080930)
Supplement: Supplementary file 1 [file cells-08-00930-s001.pdf]

Supplementary information Table 1. Results of computational prediction for HDAC3 tyrosine phosphorylation

Residue	Amino acid	Context	Score*	Kinase
7	Y	KTVA**Y**FYDP	0.398	INSR
9	Y	VAYF**Y**DPDV	0.411	INSR
18	Y	GNFH**Y**GAGH	0.46	EGFR
39	Y	LVLH**Y**GLYK	0.367	Src
42	Y	HYGL**Y**KKMI	0.405	INSR
51	Y	VFKP**Y**QASQ	0.435	INSR
66	Y	HSED**Y**IDFL	0.979	unsp
107	Y	FCSR**Y**TGAS	0.555	EGFR
146	Y	SGFC**Y**VNDI	0.58	unsp
160	Y	ELLK**Y**HPRV	0.395	INSR
166	Y	PRVL**Y**IDID	0.814	unsp
182	Y	QEAF**Y**LTDR	0.467	EGFR
195	Y	SFHK**Y**GNYF	0.455	INSR
198	Y	KYGN**Y**FFPG	0.477	INSR
207	Y	TGDM**Y**EVGA	0.739	unsp
216	Y	ESGR**Y**YCLN	0.681	unsp
217	Y	SGRY**Y**CLNV	0.428	INSR
232	Y	DDQS**Y**KHLF	0.452	INSR
247	Y	VVDF**Y**QPTC	0.44	unsp
282	Y	ECVE**Y**VKSF	0.831	INSR
309	Y	RCWT**Y**ETSL	0.643	unsp
325	Y	EELP**Y**SEYF	0.769	unsp
328	Y	PYSE**Y**FEYF	0.481	Src
331	Y	EYFE**Y**FAPD	0.507	Src
354	Y	NSRQ**Y**LDQI	0.391	INSR
387	Y	DLLT**Y**DRTD	0.366	INSR
404	Y	PEEN**Y**SRSG	0.982	unsp

The protein sequences of HDAC3 (Genebank Accession: AAC52038) was submitted in FAST format, checked for tyrosine residues for prediction, and the results displays only the best prediction for each residue.

* The true phosphorylation sites should obtain a score close to 1.0 and non-phosphorylation sites should get scores close to 0.0.

Supplementary information Table 2. Antibodies used for WB and IP assays

	Vendor Cat no.	Local	Country	Titer	usage
**αMyc**	Abcam G077	Richmond, BC	CANADA	1:1000 (1:100)	W.B (IP)
**αFlag**	Sigma-Aldrich F7425	St. Louis, MO	USA	1:1000 (1:100)	
**αHA**	Proteintech 66006-1	Rosemont, IL	USA	1:1000 (1:100)	
**αc-Src**	Cell signaling 2109	Danvers, MA	USA	1:1000	
**αEGFR**	Santa Cruz Biotechnology SC-03	Dallas, Texas	USA	1:1000	
**αp-EGFR**	Epitomics 1727-1	Burlingame, CA	USA	1:1000	
**αHDAC3**	Cell signaling #2632	Danvers, MA	USA	1:1000	
**αβActin**	Sigma-Aldrich A5441	St. Louis, MO	USA	1:10000	
**αpHDAC3$^{Y328/331}$**	AB Frontier	Seoul, South Korea	KOREA	1:2000	
**αHDAC1**	Cell signaling #2062	Danvers, MA	USA	1:1000	
